# Comparing the effect of a lactation-specific relaxation and visualisation intervention versus standard care on lactation and mental health outcomes in mothers of very premature infants (the EXPRESS trial): study protocol for a multi-centre, unmasked, randomised, parallel-group trial

**DOI:** 10.1186/s13063-022-06570-9

**Published:** 2022-07-29

**Authors:** Ilana Levene, Jennifer L. Bell, Christina Cole, Kayleigh Stanbury, Frances O’Brien, Mary Fewtrell, Maria A. Quigley

**Affiliations:** 1 National Perinatal Epidemiology Unit, Nuffield Department of Population Health, University of Oxford, Oxford, UK; 2 Newborn Care, John Radcliffe Hospital, Oxford University Hospitals NHS Trust & Faculty of Clinical Medicine, University of Oxford, Oxford, UK; 3 UCL Great Ormond Street Institute of Child Health, London, UK

**Keywords:** Newborn, Preterm, Breastfeeding, Breast milk, Maternal stress, Meditation, Relaxation therapy

## Abstract

**Background:**

Premature birth is the leading cause of neonatal death and can cause major morbidity. Maximising the amount of maternal breastmilk given to very premature infants is important to improve outcomes, but this can be challenging for parents. Parents of infants receiving neonatal care also have high rates of anxiety and distress. There is growing evidence for the impact of maternal relaxation interventions on lactation, as well as mental health. The trial will assess whether a brief self-directed relaxation and visualisation intervention, recommended for use several times a day during expression of milk, improves lactation and mental health outcomes for mothers of very premature infants.

**Methods:**

Multi-centre, randomised, controlled, unmasked, parallel-group trial with planned 132 participants who have experienced premature birth between 23 weeks and 31 weeks and 6 days of gestation and plan to express milk for at least 14 days. The primary outcome is the highest 24-h expressed milk yield recorded on any of day 4, day 14 or day 21 after birth. Secondary outcomes include exclusive breastmilk feeding at 36 weeks post-menstrual age and at 4 months after the estimated date of delivery, Spielberger State Trait Anxiety Index at day 21 and Post-traumatic stress Check List (for DSM 5) at day 21.

**Discussion:**

Breastmilk feeding for premature infants is an important research priority, but there are few randomised controlled trials assessing interventions to help parents reach lactation goals in this challenging context. This trial will assess whether a no cost, easily scalable relaxation tool has a role in this setting. Given the lack of harm and potential for immediate dissemination, even a small benefit could have an important global impact.

**Trial registration:**

ISRCTN16356650. Date assigned: 19/04/2021.

**Supplementary Information:**

The online version contains supplementary material available at 10.1186/s13063-022-06570-9.

## Background

There are an estimated 2.4 million infants born at less than 32 weeks gestation globally each year [1], and the rate of premature birth is increasing [2]. Complications arising from premature birth are the leading cause of neonatal death in the UK [3] and globally [2]. Premature infants also have increased risk of long-term disability, which increases as the gestational age at birth decreases [4]. Premature birth has a significant effect on parental mental health—systematic review estimates that 42% of parents have significant anxiety and 40% have significant post-traumatic stress reactions in the first month after birth [5]. Between 29 and 40% of mothers have depression in this timeframe [6]—all of these figures are higher than found after term, healthy birth [5, 6].

Infants born at less than 32 weeks gestation cannot fully orally feed from birth and are therefore given nutrition intravenously and/or directly into the stomach with an enteral tube. To provide breastmilk for their infants, mothers must express milk from the breasts for a prolonged period. Maximising the volume of maternal breastmilk given to these infants is associated with improved mortality, morbidity and long-term neurodevelopmental outcome [7–10]. Increasing the amount of breastmilk that mothers of very premature infants can express in the weeks after birth also leads to longer duration of breastmilk feeding [11, 12], with consequent well-known public health benefits for both infant and mother [13]. Breastmilk feeding is the provision of breastmilk by any route (for example, directly at the breast, by gastric tube or by bottle).

Despite motivation to provide expressed breastmilk for their infants, there is a high risk of poor milk supply, leading to non-exclusive breastmilk feeding [14–18] and an increasing failure to meet mothers’ own goals for breastmilk provision as time goes on [19]. It is challenging to establish and then maintain a full milk supply through exclusive expressing [20, 21], which is exacerbated when pregnancy has been abbreviated and the breast tissue and hormonal milieu are therefore not at an optimal stage. Perinatal interventions associated with premature birth may also pose lactation challenges—such as provision of antenatal steroids, which also delay milk ‘coming in’ [22] (lactogenesis II), and caesarean birth.

Although breastmilk feeding in prematurity has been identified as a top ten research priority by the James Lind Alliance priority setting partnership [23], there are few randomised controlled trials (RCTs) related to breastmilk expression [24]. A Cochrane review noted that one promising technique to improve expressed milk yield is guided relaxation and visualisation by the mother and recommended further research in this area [24]. Several neonatal unit RCTs [25–28] have shown increased expressed milk yield after a relaxation intervention, some with a dramatic effect (doubling the average yield) [25]. Studies in mothers of healthy babies have also shown reduced time to lactogenesis II [29], markers of improved milk transfer (such as infant weight gain) and reduction in maternal stress and anxiety [30, 31].

The theoretical basis of the effect of relaxation and visualisation on lactation is threefold—directly through the interaction of stress and lactation hormones, directly through the central nervous system and indirectly through behavioural factors. The major hormones of milk production and release are prolactin and oxytocin, which are closely connected to the wider web of hormones influencing perinatal mood [32]. For example, salivary cortisol is associated with prolactin level in mothers of very premature babies over 6 weeks of expressing milk [33] and measures of anxiety such as the State-Trait Anxiety Index (STAI) correlate with oxytocin level at 8 weeks after birth [34]. Relaxation interventions can reduce salivary and breastmilk cortisol [30, 35]. Thus it is possible that relaxation affects hormones such as cortisol, which then affect prolactin and oxytocin level.

Mental visualisation can trigger the milk ejection reflex (MER—the muscular ejection of milk), as seen in mothers with high spinal cord injury who can trigger MER with visualisation routines in the absence of any intact sensory pathways from the breast [36]. Mothers of premature infants often report difficulties with MER due to the reduced physical contact with their babies. Inhibition of MER leads to incomplete drainage of the breast and therefore secondary inhibitory feedback of milk production [37]. Targeting MER through visualisation therefore has the potential to increase milk yield.

Lastly, there may be behavioural mediators, whereby mothers who have less perception and manifestation of stress are more likely to interact positively with their infant [38] and have increased self-efficacy [39]. They may therefore put in place a more effective regime of frequent expressing and perform other actions with a positive influence on lactation such as spending more time in skin-to-skin contact with their infant.

A key weakness of existing studies is the focus on short-term outcomes, which in several cases have minimal clinical significance [26, 27, 35, 40]. There are also significant concerns over risk of bias in many of the studies reported, including inadequate allocation concealment [26, 27], insufficient washout period for a crossover study [35] and selective reporting [27, 29, 41].

## Methods

### Aim and design

This is a multi-centre, unmasked, randomised controlled, parallel-group trial designed to determine if a self-directed relaxation and visualisation intervention for mothers of infants born between 23 weeks of gestation and 31 weeks and 6 days of gestation improves lactation and mental health outcomes. Both groups will receive standard clinical support for lactation provided by their neonatal unit staff. The intervention is a 12-min-long modified audio recording that participants are requested to listen to frequently while expressing breastmilk. The primary objective is outlined in Table 1.

**Table 1:** Primary objective in PICO format

	Primary objective
**Population**	Mothers of one or two infants born between 23+0 and 31+6 weeks’ post-menstrual age
**Intervention**	Provision of specific relaxation and visualisation recording with instruction to listen frequently while expressing breastmilk
**Control**	Standard care, including standard lactation support provided by neonatal unit staff
**Outcome**	Expressed milk yield in 24 h (highest value recorded on any of day 4, day 14 or day 21 after birth)

**Fig. 1:**
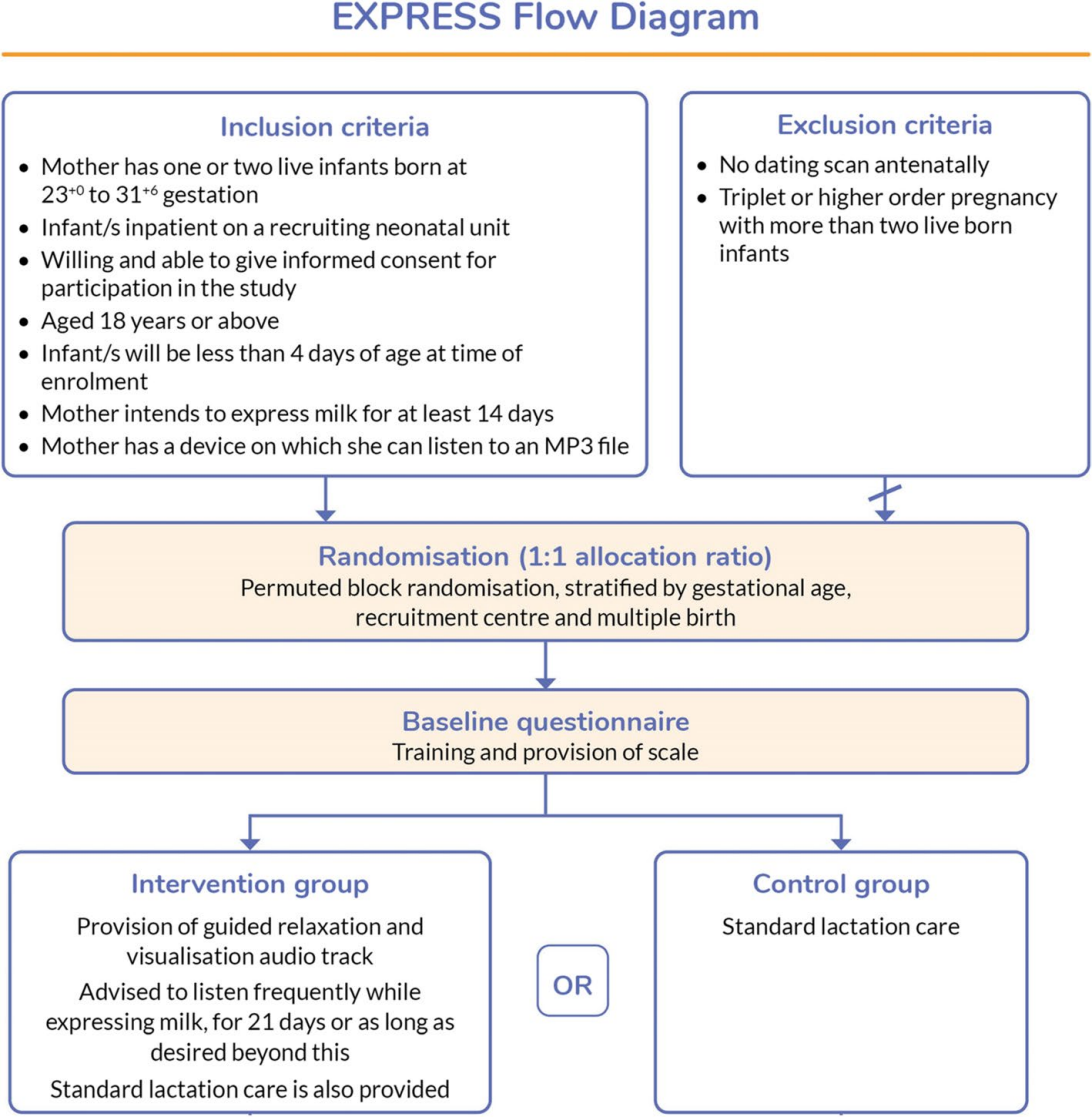
Trial summary flowchart

Data is submitted directly by participants into case report forms and through participant responses to short message service (SMS) messages. Research staff enter baseline and secondary outcome data from the participant and infant medical notes. The trial aims to recruit a total of 132 participants. The trial design is summarised in Fig. 1.

**Fig. 2:**
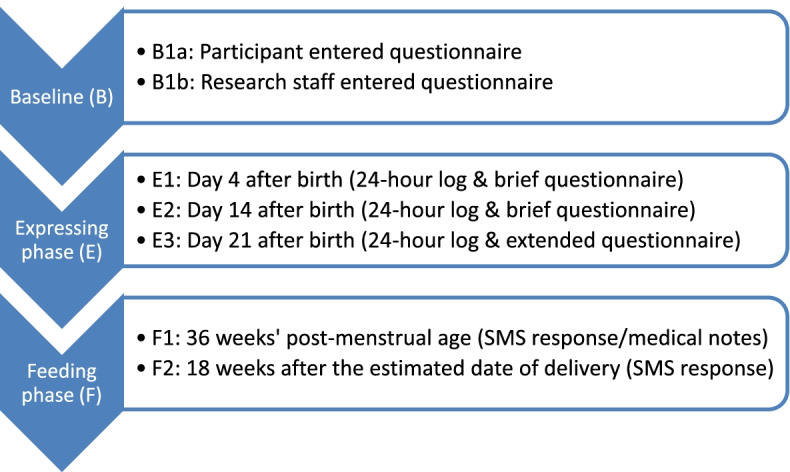
Timepoints for trial data collection

Data will be collected at six timepoints, described in Fig. 2 and Table 2. At baseline, the participant completes a case report form (CRF) questionnaire about themselves, their previous breastmilk feeding experience and feeding intentions (B1a). Research staff complete a trial entry CRF with information about the birth and infant’s medical status at randomisation (B1b). In the expressing phase of data collection, the participant completes a CRF questionnaire and 24-h log of expressed milk at three timepoints. E1 is day 4 after the infant’s birth (the day of birth is termed day 0). E2 is day 14 and E3 is day 21 after the infant’s birth. In the feeding phase of data collection, the participant responds to an SMS message asking about the infant’s feeding status, at two timepoints. F1 is at 36 weeks’ post-menstrual age (PMA; 4 weeks before the estimated date of delivery—the EDD—which defines 40 weeks of gestation) and F2 is at 18 weeks after the EDD—their SMS response automatically completes a CRF for each timepoint. If no SMS is received, infant feeding status can be extracted from the medical notes.

**Table 2:** Schedule of trial procedures

Procedures	From the day of birth (day 0) up to midnight on day 3 of life	Day 4, 14 (E1 & E2)	Day 4 to 7	Day 21 (E3)	32 weeks post-menstrual age ***(exploratory timepoint)***	36 weeks post-menstrual age (F1), 9 and 18 weeks after estimated date of delivery (F2)
					*Only applies if infant born at <27 weeks gestation*	*In the abbreviated follow-up period the trial completes at F1*
Informed consent	✔					
Eligibility assessment	✔					
Randomisation	✔					
Baseline Questionnaire	✔					
Maternal training and assessment on use of scales	✔					
Check-in and verify accurate use of scales			✔			
Questionnaire & 24-h expressing log		✔		✔		
Maternal mental health questionnaires				✔		
Text message response					✔	✔

Two further exploratory data collection timepoints will take place, which do not contribute data to the RCT, but to exploratory analyses. These are a 24-h expressing log and questionnaire CRF at 32 weeks PMA, for participants who had given birth at less than 27 weeks PMA; and a feeding assessment SMS message at 9 weeks after the EDD. For example, an infant born at 24 weeks of gestation is 8 weeks old at 32 weeks PMA and 25 weeks old at 9 weeks after the EDD.

The trial forms part of a doctoral thesis. For reasons of efficiency within the available time, the recruitment period is divided into two sections. The majority of recruitment (10 months) will take place in the ‘complete follow-up’ period where participants will receive both F1 and F2 SMS messages. The final 22 weeks of recruitment will form the ‘abbreviated follow-up’ period where participants will receive only the F1 SMS message.

**Fig. 3:**
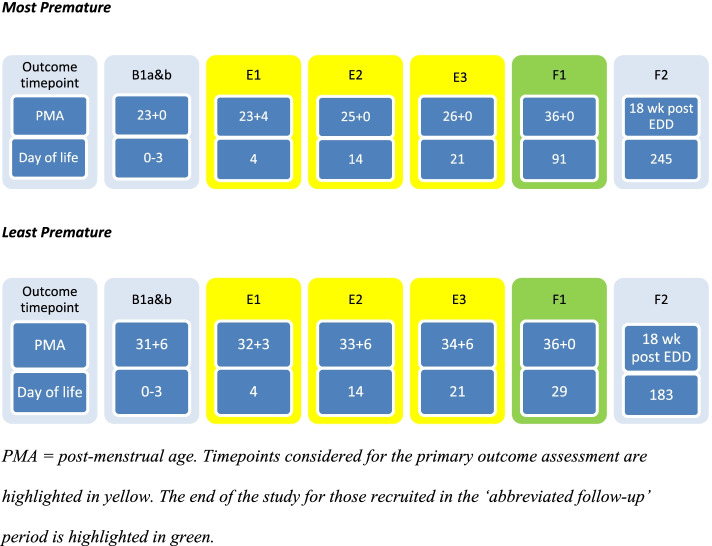
Sample timeline for most and least premature infant eligible

The time from recruitment to the end of the expressing phase of the study is 3 weeks. The time from recruitment to the end of the feeding phase of the study varies with the gestation of the infant and the follow-up period. The longest possible time—for a participant recruited in the ‘complete follow-up’ period with an infant born at 23 weeks—will be 35 weeks. The shortest possible time—for a participant recruited in the ‘abbreviated follow-up’ period with an infant born at 31 weeks and six days—will be 4 weeks. Figure 3 shows sample timelines for the most and least premature infants.

### Setting and participants

The trial plans to recruit participants within three National Healthcare Service Trusts in northern and southern England, covering four individual neonatal units (three tertiary level intensive care units and one local neonatal unit). All sites have at least one dedicated infant feeding support professional and counselling or psychological support is available. All sites have hospital-grade pumps available for use in the hospital and a loan scheme for free use at home. Breastmilk feeding rates at discharge are variable, with one site below the regional and national averages and the others above average [42]. Two Trusts have level 3 Unicef Baby Friendly Initiative accreditation and one has not yet started the accreditation process. All sites are urban and serve a range of communities, including diversity of ethnic origin and social deprivation.

Participants are mothers of infants born between 23^+0^ and 31^+6^ weeks of gestation. Full inclusion and exclusion criteria are provided in Fig. 1. Participants with more than two live born infants are excluded and participants must have an intention to express milk for at least 2 weeks after birth to join the trial. Participants can be screened by research nurses and trained clinicians during pregnancy and after birth. Participants must provide written, informed consent (either on paper or electronically) and are provided with written information sheets. Randomisation can only take place between birth and midnight on day 3 after birth.

### Description of intervention

The intervention is the provision of a specific recording to the participant as an MP3 file with a request to listen frequently during expression of milk, on their own device, for at least 3 weeks. The intervention recording lasts approximately 12 min and consists of a guided relaxation and expression-specific visualisation. This is a modified version of an existing soundtrack used for previous studies [25, 30, 43, 44], modified and used under licence from the original author. The visualisation includes descriptions of pleasant surroundings, milk flowing in the breasts and skin-to-skin contact with the infant. The modified version will be made freely available after the trial is complete.

Both intervention and control groups will receive general lactation care, including routine advice from neonatal unit staff and standardised printed information on milk expression complying with Unicef Baby Friendly Initiative recommendations [45].

### Randomisation

Participants will be randomised with a 1:1 allocation using stratified permuted block randomisation by a secure web-based randomisation programme hosted by the National Perinatal Epidemiology Unit Clinical Trials Unit. Stratification will be based on recruitment neonatal unit, gestational age at birth (23^+0^ to 27^+6^ weeks versus 28^+0^ to 31^+6^ weeks) and multiple birth (one versus two babies alive at time of randomisation). Research staff and the participant are aware of the allocation after randomisation, as the study is unmasked. Study identification numbers are not consecutive, to reduce the possibility of research staff monitoring the sequence of allocation.

### Primary outcome

The primary outcome is defined as the highest 24-h expressed milk weight recorded on any of day 4, day 14 or day 21 (E1, E2 or E3; in grams). If the participant declares that they are no longer expressing, the milk weight for that day is accorded a value of zero. Table 3 shows various scenarios of what this means in practice. The reason for this definition of the primary outcome, instead of using 24-h expressed milk weight at a specific timepoint such as day 21, is that some more mature infants may start to directly breastfeed by day 21 and this is likely to decrease expressed milk yield. In addition, some parents may choose to stop expressing before day 21 but increasing their milk yield prior to this would still be an important outcome to reduce infant morbidity. Finally, the physiology of lactation studied in term infants [46] suggests that once a milk supply is established, it is robust to supply and demand—therefore, achieving a higher milk yield at day 14 than day 21 suggests that the parent will be able to increase their yield as needed with more frequent expressing in the future.

**Table 3:** Examples of primary outcome calculation

	Day 4	Day 14	Day 21	Primary outcome
**Scenario 1**	300g	500g	750g	750g
**Scenario 2**	100g	450g	400g	450g
**Scenario 3**	50g	missing	missing	50g
**Scenario 4**	50g	150g	Stopped expressing (0g)	150g

### Secondary outcomes

The following secondary outcomes will be reported:

- Proportion of participants expressing at least 750g of milk in 24 h on any of day 4, 14 or 21 (E1, E2 or E3)
- Rate of milk expression (in grams per minute) at day 21 (E3)
- Average Spielberger State Trait Anxiety Index (six-item format; STAI-6) score at day 21 (E3)
- Average Post-traumatic Stress Checklist for DSM-5 (PCL-5) score at day 21 (E3)
- Proportion of participants feeding their infant/s only breastmilk (no infant formula) at 36 weeks PMA (F1)
- Proportion of participants feeding their infant/s any breastmilk at 36 weeks PMA (F1)
- Proportion of participants feeding their infant/s only breastmilk (no infant formula) at 18 weeks after the estimated date of delivery (4 months corrected age—F2)

The STAI is a reliable measure of anxiety [47] and widely used, including in studies after very premature birth [48, 49] and after use of a relaxation intervention in the neonatal intensive care unit [27]. The original STAI is a 20-item questionnaire with four answer options for each question (‘not at all’, ‘somewhat’, ‘moderately’ or ‘very much’). The total score range is 20–80, with higher scores signifying more anxiety, <36 generally considered normal [50] and ≥40 signifying clinically significant anxiety [48] (although this cut off varies across the literature [5]). The shortened STAI-6 contains a subset of six questions, giving a score total of 6–24, which are then scaled to 20–80 for comparability [51]. The short form is highly correlated with the 20-item STAI, with internal consistency greater than 0.9 [52]. The minimal meaningful difference in STAI score has been suggested as 10 [53].

The PCL-5 is a commonly used [54] publicly available screening assessment for post-traumatic stress disorder (PTSD) aligned to the Diagnostic and Statistical Manual of Mental Disorders-Fifth Edition (DSM-5) [55]. It has been used in the context of birth related trauma [56]. The PCL-5 is very similar to the Impact of Event Scale (IES) and the preceding PCL for DSM-IV that have both been used in the specific context of premature birth [48, 57]. The PCL-5 [55] has 20 items with four answer options for each question (‘not at all’, ‘a little bit’, ‘moderately’, ‘quite a bit’ and ‘extremely’). The total score range is 0–80, with higher scores signifying more distress. A cut off of 31–33 [58, 59] has been proposed as indicative for probable PTSD. A 5–10 point change is likely to be the minimum threshold for a clinically meaningful difference [55]. The PCL-5 has internal consistency (for example, Cronbach’s alpha 0.95) and good construct validity in a variety of settings [58–60]. Of note, a diagnosis of PTSD requires symptoms to continue beyond 28 days after the traumatic event—therefore, in this context, the PCL-5 will measure post-traumatic stress reactions rather than PTSD.

In relation to the secondary outcomes measured at 36 weeks PMA and 4 months corrected age (F1 and F2), note that no reference is made to whether the infant is having complementary feeds (‘solids’) in addition to milk. This is because the timing of introduction of complementary feeds for premature babies is not recommended by actual age as it is with term babies, but rather personalised to the developmental stage, actual and corrected age of the infant [61].

### Process indicators

The following process indicators will be reported:

- Time spent in skin to skin contact with infant/s at day 21 (E3; in hours)
- Number of expressing episodes in 24 h at day 21 (E3)
- Total time spent expressing in 24 h at day 21 (E3; in hours)

The process indicators were chosen as they may indicate a behavioural pathway for any effect seen on the primary outcome.

### Monitoring adherence and contamination

At E1, E2 and E3, participants in the intervention group are asked how many times they have listened to the intervention in the previous 24 h. At E3, both groups are asked how often they have practised other forms of relaxation since the birth of their infant/s.

### Measures to improve data quality and retention

Research nurses and trained clinicians will have close contact with participants during the expressing phase. At baseline, participants are given written information on weighing milk and using the electronic forms; short videos are also available. Research staff contact participants after the first expressing log (E1) to ensure that the weighing record is accurate and respond to questions. Automated reminder emails and SMS messages are sent to participants if forms are not filled in. Trial staff check participant-submitted forms and research staff raise any obvious problems with participants in a timely period. Automated SMS messages are sent to the intervention group on day 9 and day 17 after birth to remind them to listen to the intervention. Prior to the feeding phase assessments (F1 and F2), emails are sent explaining their importance.

### Withdrawal and change in consent

Each participant has the right to change their consent at any time and will not be contacted for further data submission after this time. In addition, the Investigator may withdraw a participant from the study at any time if the Investigator considers it necessary for any reason including:

- Ineligibility (either arising during the study or retrospectively having been overlooked at screening)
- Death of the mother or the infant/s during the study period

The number of participants withdrawn and changing their consent status will be reported by trial arm, with reasons. Participants choosing to stop listening to the intervention recording but continuing follow-up will remain in the study. Participants may choose to continue in the study after the death of a single twin.

### Data collection

At E1, E2 and E3, participants will be asked to record information about each time that they attempt to express milk in a 24-h period, starting when they wake up for the day. Participants will be trained at recruitment to weigh their expressed milk inside its container and to record the weight of the empty container. At analysis, the pure milk weight expressed can therefore be calculated and summed over the 24-h period. Each participant will be given a portable digital scale accurate to 0.1g (Kabalo). Participants can fill in the expressing log and questionnaire on paper or on an electronic form with individual URL, hosted by OpenClinica (a third party application hosted in the UK by an ISO 27001:2013 accredited third party; AA). Paper forms can be sent by post or photographs can be uploaded securely by participants. Participants will receive automated notifications the night before and on the morning of the scheduled day, and a reminder the next day if appropriate. The scheduled period for data response is within 48 h of the scheduled day.

Participants can express milk by whatever method they desire. By day 4 after birth (E1), all sites recommend participants to use a double hospital-grade electric pump.

At F1 and F2, participants are sent an SMS message asking what type of milk their infant/s is drinking—only breastmilk, only infant formula or a mixture of the two. The scheduled period for data response is within 7 days of the respective timepoints. At F1, infant feeding status can be extracted from medical notes if no response is received.

Basic demographics of the potentially eligible population will be extracted from routinely entered clinical data in the BadgerNet platform by clinical staff and fully anonymised before transfer to the NPEU, as a screening log.

No formal safety reporting is required due to the low risk nature of the intervention.

### Project management

The trial will be run on a day-to-day basis by the Project Management Group (PMG), which reports to the Trial Steering Committee (TSC). The TSC receives recommendations related to the data arising from the trial from the Data Monitoring Committee (DMC). The PMG will consist of the Chief Investigator, Clinical Trials Unit Director, Head of Operations, Senior Trials Manager, Trial Programmers, the Trial Statistician and Trial Administrator. The PMG will meet every 3 to 4 weeks. The TSC and DMC will consist of independent clinical and statistical experts, as well as parent and public representatives. The TSC and DMC will meet three times over the course of the trial; charters are available by contacting the study team. Both are independent from the sponsor. The trial will be run according to the Clinical Trials Unit standard operating procedures, including potential audit. The sponsor is the University of Oxford (ctrg@admin.ox.ac.uk), which has ultimate authority over the trial’s scientific integrity.

### Patient and public involvement

National charity for sick and preterm babies, Bliss, has been a co-applicant for the study since its inception. A total of 675 people who had experience of premature birth were involved in initial online engagement work to define the most important outcomes for the RCT, give their thoughts on ethical issues and share the ways in which they found information about lactation to inform the dissemination plan. From this pool of parents, six also attended virtual panels to give detailed feedback on trial design and participant-facing documents, and a further 12 took part in an exercise to advise on modifications to the intervention recording. Modifications were made to the intervention recording in three key areas raised by parents—minimising a feeling of pressure on volumes of milk expressed, sensitive language related to potentially traumatic birth and language appropriate to immature and potentially sick infants. Bliss and a parent contributor are members of the Trial Steering Committee.

### Statistics and analysis

#### Sample size and power calculation

In the most relevant previous study [25], mean expressed milk yield at day 14 of the trial increased from 318 ml (standard deviation; SD 309 ml) to 862 ml (SD 309 ml) with the use of a visualisation/relaxation soundtrack. Initial exploratory work suggested that baseline milk yield in the recruiting sites is higher than this, and therefore, this study is powered to detect an increase in highest 24-h expressed milk yield from 670g (SD 300g) to 825g (SD 300g), with 80% power and a two-sided significance level of 0.05. This is a 155g absolute mean difference or a 0.5 standardised mean difference. In total, 118 participants are required, so the recruitment target was set at 132 to allow for 10% attrition. Since breastmilk has an average specific gravity of 1.03 [62, 63], 670g is estimated as equivalent to 650ml and 825g is estimated as equivalent to 800ml, although much of the literature views volume and mass measurements as interchangeable for breastmilk [64–66].

#### Description of statistical methods

Demographic and clinical data will be summarised with counts and percentages for categorical variables, means (with SDs) for normally distributed continuous variables and medians (with interquartile or simple ranges) for other continuous variables.

Statistical analysis of continuous outcomes will use linear regression with adjusted and unadjusted mean differences presented, or quantile regression with median differences presented, as appropriate. Binary outcomes will be analysed using log binomial regression, or Poisson regression with a robust variance estimator if the model fails to converge, and risk ratios will be presented. 95% confidence intervals will be presented.

Analyses will be adjusted for the stratification factors (recruiting neonatal unit, gestational age at birth and whether the participant has one or two babies alive at randomisation) where possible. The STAI score at day 21 will be adjusted for the STAI score at baseline. The primary outcome will be adjusted for the associated measurement day of the highest milk weight. Both crude and adjusted estimates will be presented, but the primary inference will be based on the adjusted estimates. Further process indicators, such as number of direct breastfeeds, will be summarised by randomised arm, with no comparative statistics presented.

Participants will be analysed in the groups to which they were randomly assigned, comparing the outcome of all participants allocated to intervention with all those allocated to the comparator group, regardless of deviation from the protocol or treatment received (the Intention to Treat population). Analysis will take place on a superiority basis.

Exploratory subgroup analysis will use a statistical test of interaction to examine the effect of the intervention on the primary outcome by gestational age at birth (<28 weeks and ≥ 28 weeks). Stratified analysis by the other stratification factors described and exclusive breastmilk feeding intention at baseline will take place with no formal test for interaction. This is to limit multiple comparisons and due to the low statistical power for subgroup analysis.

An exploration of adherence effect on the primary outcome will be conducted, dividing the intervention group into high- and low-frequency adherence by their reported number of times listening to the recording. An exploration of perceived relaxation effect on primary outcome will be conducted, dividing the intervention group into those who report the intervention to be relaxing and those who do not (via Likert scale). Summary statistics will be presented, with no statistical inference.

Basic demographics of the recruited participants will be compared with aggregate data from the potentially eligible population with no statistical inferences made.

Primary analysis will take place on a complete case basis. If more than 10% of primary outcome data is missing, sensitivity analysis will be performed to explore the pattern of missingness for the primary outcome, using multiple imputation and/or a pattern mixture model as appropriate. Sensitivity analyses will also assess whether the definition of the primary outcome has led to bias by assessing the impact of using day 21 expressing yield only, and separately of excluding day 4 logs. Stata will be used for all analysis.

### Participant confidentiality and retention of personal data

The Participant Information Sheet provides the required information about data processing. Participant contact details are necessary to send CRFs and SMS messages. These will be kept in an administrative database, linked to the clinical database by study identifier. All electronic databases comply with relevant security standards and are regularly backed up. Identifiable information will be kept centrally for a period of 5 years and then reviewed. All data kept centrally will be archived according to Clinical Trials Unit standard operating procedures.

### Participant remuneration

Each participant will be provided with a small digital weighing scale, a coolbag and a pen. They will be offered a set of headphones if in the intervention group. Due to higher than expected loss to follow-up for the primary outcome, an amendment was approved after recruitment started to provide a £10 electronic food and drink voucher code to participants during the expressing phase. This is an unconditional thank you gift for the participant with the aim to increase the participant’s feeling of connection with and positivity about the trial.

### SPIRIT guidelines

This protocol report uses the SPIRIT reporting guidelines [67]. The SPIRIT checklist is provided as supplementary material (Additional file 1).

## Discussion

### Ethical considerations

The trial will run in the context of families experiencing the trauma of very premature birth. Many babies will be very unwell, or perceived to be very unwell by their families even when their clinical course is smooth. Some babies may die, including one of a multiple birth where the other baby survives. Much of the recruitment period will take place at a time when parents are likely to be further affected by the Covid-19 pandemic, both in their lives outside of the neonatal unit and in terms of potential increased separation from and anxiety about their baby. The potential participants of the trial are therefore facing many ethical crises and are under major stress throughout the period of the trial. The trial team has collaborated with parent panels and national charity for sick and premature babies, Bliss, to ensure that the trial structure and the language of all trial documents take this situation into account. All participant-facing CRFs include links for more information about having a premature baby and expressing milk. Free text questions are included to allow participants to express their feelings and feel seen as individuals. Infant deaths will be frequently monitored during the trial period to ensure we are not asking mothers for research data at the time of their bereavement. Staff will be encouraged to undergo bereavement training.

If the participants’ scores on the mental health assessments at day 21 exceed a pre-specified threshold for concern, the participant will be sent information on self-help and how to access individualised neonatal unit-based psychological support.

### Dissemination

A dissemination plan has been developed, using the parent involvement process to identify key sources of information about lactation. The trial findings will be disseminated via academic conferences and publications, to clinical staff via clinical networks and to parents via social media and online support networks as well as printed information distributed to neonatal units. There are no plans to use professional writers and all authors will fulfil authorship criteria.

### Impact and risk of bias

This study will report on whether a simple, easily disseminated relaxation intervention improves expressed milk yield, duration of breastmilk feeding and mental health outcomes for mothers of very premature infants. The intervention recording will be made freely available after the results are reported, so if proven beneficial, there will be an immediate positive impact on parents globally, with the caveat of the English language nature of the recording. A strength of this trial is the robust design with full Clinical Trials Unit support, which is unusual for RCTs in the field of lactation. Further strengths include extensive parent involvement, a nuanced clinically important primary outcome and pre-specified sensitivity analyses to assess potential bias.

Study outcomes will be broadly generalisable to parents of infants born at less than 32 weeks of gestational age in countries with advanced neonatal care, and are likely to apply to parents expressing milk for sick infants of all gestations as well. Inclusion criteria are broad to recruit a representative population, not just those who are highly motivated to express and exclusively breastfeed, which increases external validity. It is possible that the effect of the intervention will depend on the level of wider support available to the participants and the trial includes neonatal units with a range of lactation support intensity.

There is likely to be some ‘trial effect’ reducing external validity—it is known that the use of expressing logs and increased focus on expressing characteristics can improve adherence to recommendations and therefore may improve outcomes across all participants, including the control group [68]. However, there is no way to overcome these limitations while robustly studying expression characteristics.

There is inherent potential for bias in all trials where the intervention cannot by its nature be blinded. Participants in the intervention group may express more frequently or for longer periods of time in order to please the research team or because they believe in the power of the intervention. The study is designed to report process indicators to see if this has taken place (although these factors are also possible behavioural mechanisms for an intervention affect, rather than necessarily a sign of bias). Techniques of partial blinding due to deception used in other trials [30, 43, 44] (where the control group are not aware of the existence of the intervention) are not appropriate where participants interact with each other frequently. It is therefore acknowledged that the study will have some unmodifiable risk of bias. Nevertheless, it will be a worthwhile contribution to understanding lactation in challenging circumstances.

### Trial status

Recruitment began 2 August 2021. Recruitment completion will be 31 October 2022. Current protocol is version 5.0 26/05/2022. Amendments are submitted to the Research Ethics Committee and then disseminated as recommended by the committee.

## Supplementary Information

**Additional file 1.** SPIRIT Checklist. Completed SPIRIT Checklist as per journal requirement.

## Data Availability

The authors welcome requests from researchers to access data held by the Nuffield Department of Population Health (NDPH). Access to the datasets will be controlled by the data custodian and subject to further regulatory approvals. The NDPH Data Access policy sets out the criteria and process for data sharing. Requests for data sharing that meet the criteria will be considered by the NDPH data sharing committee. The criteria are as follows: to ensure the integrity of any data that it releases to the public (e.g. resolving data quality issues); and to allow a reasonable period of exclusivity. The committee will assure themselves that the proposal is scientifically sound, the protocol has been peer reviewed, there is adequate funding and there are appropriate ethics approvals in place. The committee will require evidence that the likely commitment of NDPH staff has been realistically assessed and will normally expect funding to cover any NDPH costs in making data available and providing ongoing support. The committee must also assure itself that releasing the data can be justified within the scope of the original participant consent. Shared data will be anonymous. The following information is given in the Participant Information Sheet for this study ‘Anonymised information from this study may be shared with other researchers doing similar research in the future. None of your personal identifiable information will be shared, and you will not be identifiable from this data’.

## Abbreviations

CRF: Case report form
DMC: Data Monitoring Committee
DSM: Diagnostic and Statistical Manual of Mental Disorders
EDD: Estimated date of delivery
MER: Milk ejection reflex
PCL: Post-traumatic Stress Checklist
PMA: Post-menstrual age
PMG: Project Management Group
PTSD: Post-traumatic stress disorder
RCT: Randomised controlled trial
SD: Standard deviation
SMS: Short messaging service
STAI: Spielberger State Trait Anxiety Index
TSC: Trial Steering Committee
